# Impact of Ramadan Fasting on Dietary Intakes Among Healthy Adults: A Year-Round Comparative Study

**DOI:** 10.3389/fnut.2021.689788

**Published:** 2021-08-05

**Authors:** Hibeh Shatila, Mariam Baroudi, Raeda El Sayed Ahmad, Rana Chehab, Michele R. Forman, Nada Abbas, MoezAlIslam Faris, Farah Naja

**Affiliations:** ^1^Department of Nutrition and Food Sciences, Faculty of Agriculture and Food Sciences, American University of Beirut, Beirut, Lebanon; ^2^Nutrition Sciences, College of Health and Human Sciences, Purdue University, West Lafayette, IN, United States; ^3^Department of Clinical Nutrition and Dietetics, College of Health Sciences, Research Institute of Medical & Health Sciences (RIMHS), University of Sharjah, Sharjah, United Arab Emirates; ^4^Faculty of Agriculture and Food Sciences, American University of Beirut, Beirut, Lebanon

**Keywords:** fasting, dietary change, ethnic group, foodculture, intermittent fasting, Ramadan, religious affiliation

## Abstract

Religious rituals are considered among the principle factors that impact dietary behaviors and food selections. The main objective of this study is to characterize food intake among Lebanese adults observant of the fasting month of Ramadan and compare it to their intake of the rest of the year. During a year-round study, including the month of Ramadan, Lebanese adults (*n* = 62), completed multiple (9 to 13) 24-h dietary recalls. Information about sociodemographic and lifestyle characteristics was also obtained. Dietary intake was examined using food groups as well as energy, macro, and micronutrient consumption. Significant differences in dietary intakes were observed for 12 of the 19 food groups (expressed as a percent of total energy) during Ramadan as compared to the rest of the year. More specifically, the intakes of cereals, cereal-based products, pasta, eggs, nuts and seeds, milk and dairy, and fats and oils were lower, while vegetables, dried fruit, Arabic sweets, cakes and pastries, and sugar-sweetened-beverages intakes were higher during Ramadan as compared to the remainder of the year (*p* < 0.05). Such differences in food groups' intakes were reflected in nutrients intakes, including carbohydrates, cholesterol, calcium, beta-carotene, vitamin C, folate, and magnesium. The findings of this study highlighted major differences in dietary intakes between the fasting month as compared to the rest of the year. With the large number of adults who observe fasting during Ramadan, the particularities of dietary intake during Ramadan ought to be considered in the development of context and culture-specific dietary recommendations.

## Introduction

Religious affiliation is one of the most distinguishing characteristics of the world population, with Islam as the second-largest affiliation after Christianity. Many of the major religions have their unique dietary rules, which may or may not be strictly adhered to by the followers (1). In essence, religion and religious rituals and feasts are considered among the principle factors that impact dietary behaviors and food selections (2). During Ramadan, food intake shifts drastically from diurnal to nocturnal eating time and practice, Ramadan fasting illustrates how religious beliefs affect human dietary behavior.

On average, one and a half billion Muslims around the world observe the fast during the holy month of Ramadan (3). Ramadan is the ninth month of the Islamic lunar calendar when Muslims fast from dawn to sunset for 29 or 30 consecutive days, abstaining from any food or water with a common practice of consuming one large meal after dusk and a lighter meal before dawn (4, 5). Between dusk and dawn, there are no dietary restrictions related to Ramadan as it is observed in other religions like Christianity fasting (e.g., avoidance of animal-based products, oil, or fish) (6). The duration of the daily fast during this month varies according to the geographical area and to its timing in the year, reaching in some countries and seasons to 19 h a day. This yearly fast, combined with many lifestyle modifications in physical activity (7, 8), sleep patterns and circadian rhythmic changes (9–11) have been shown to incur significant changes in dietary habits and food consumption patterns (12–14) leading ultimately to significant anthropometric, cardiometabolic, glucoregulatory, and inflammatory changes (15).

The nature of food intake during the month of Ramadan, with its particularities in terms of timing and frequency of meals, has been previously examined (16–18). More specifically, food intake during Ramadan has been associated with major changes in dietary patterns, food groups as well as energy, macro-, and micronutrient intakes. However, the available evidence concerning these changes is inconclusive (13, 14, 16, 19–26). While a few studies reported lower energy intakes (18, 19, 26), several studies comparing dietary intake during Ramadan to that during regular days reported higher energy consumption during Ramadan (16, 22, 27), mainly derived from a higher intake of carbohydrates (16, 23, 26, 27), particularly sweets (14), and fats (14, 21, 22). Such changes in dietary intake during this month are of significance, especially among individuals susceptible to metabolic diseases linked to these changes such as obesity, diabetes, and hypertension (14, 28–30). Thus these food practices are important to consider in countries with a high burden of non-communicable diseases (NCD) and where considerable proportions of the populations observe fasting during Ramadan.

Lebanon is a small country in the Middle East which, according to the World Health Organization, has surging rates of NCDs, estimated to account for 91% of all deaths in the country (31). Concomitant with the high prevalence of NCDs similar to its neighboring countries, Lebanon has been undergoing a nutrition transition characterized by dietary shifts toward a surplus of energy, fat, and sugars, resulting in increasing rates of weight gain and obesity (32). Studies examining dietary patterns in the country also reported a gradual erosion of the traditional Lebanese dietary pattern rich in fruits, vegetables, bulgur, legumes, olives, whole-fat dairy, and starchy vegetables (32–37). This latter pattern was identified as a variant of the Mediterranean diet, in light of both its composition and protective effects against metabolic diseases including obesity, hypertension, and type 2 diabetes (34, 35, 38–42).

With a large proportion of Muslims (over 61%) among the Lebanese (43), and the possibly high observance of fasting during the month of Ramadan, it is critical to understand the particularities of dietary intakes during this month to develop evidence-based recommendations for healthy adults and those living with the chronic disease during the holy month.

To our knowledge, there is a paucity of research that compares the dietary intake of adults who observe the month of Ramadan with their intake during the remainder of the year. Therefore, using multiple 24 h recalls over a year, this study aims to characterize food intake among Lebanese adults observant of the fasting month in terms of energy, macronutrients, micronutrients, and food groups during the month of Ramadan in comparison with their intake during the rest of the year. The results of this study will provide an evidence base for the development of culture-specific dietary guidelines during this month in Lebanon and its neighboring countries, which share with it many cultural, religious traditions, and food-related habits.

## Methods

### Study Participants

Data for this study were derived from an earlier prospective investigation of dietary intake among Lebanese adults (18–65 years), the details of which are published elsewhere (44). In brief, subjects were recruited from the American University of Beirut (AUB), the largest employer in the Lebanese private sector (45, 46). The university is also a culturally diverse institution with employees from across the country and from various socioeconomic statuses. Flyers with invitations to participate in the study were posted on the advertisement boards across the AUB campus. The inclusion criteria were: (1) full or part-time employment at AUB, (2) Lebanese nationality, or residing in Lebanon for more than 10 years, (3) conversant in Arabic or English language, (4) not pregnant or breastfeeding, (5) not having any chronic health condition that requires dietary modifications and medication prescription. The Institutional Review Board of the Social and Behavioral Sciences at the AUB, Lebanon, approved the study protocol under the protocol number (NUT.FN.22). For this study, observant (fasting) participants of the month of Ramadan were included (*n* = 62 out of 107 who participated in the original study).

### Data Collection

Face-to-face interviews were conducted in a private setting at the participant's office or, if not available, at the Nutrition and Food Sciences department at AUB. During the 1-year duration of the study, a total of 9–12 24-h dietary recalls (24HR) were collected, at least one of which was carried out during the month of Ramadan (26th of May until 24th of June). Given that the fast period during Ramadan is related to sunset and sunrise timings, it tends to be vary from day to day. Generally, during the year where data collection took place, the fast was between 3:30 a.m. and 8:00 p.m.

**Figure 1:**
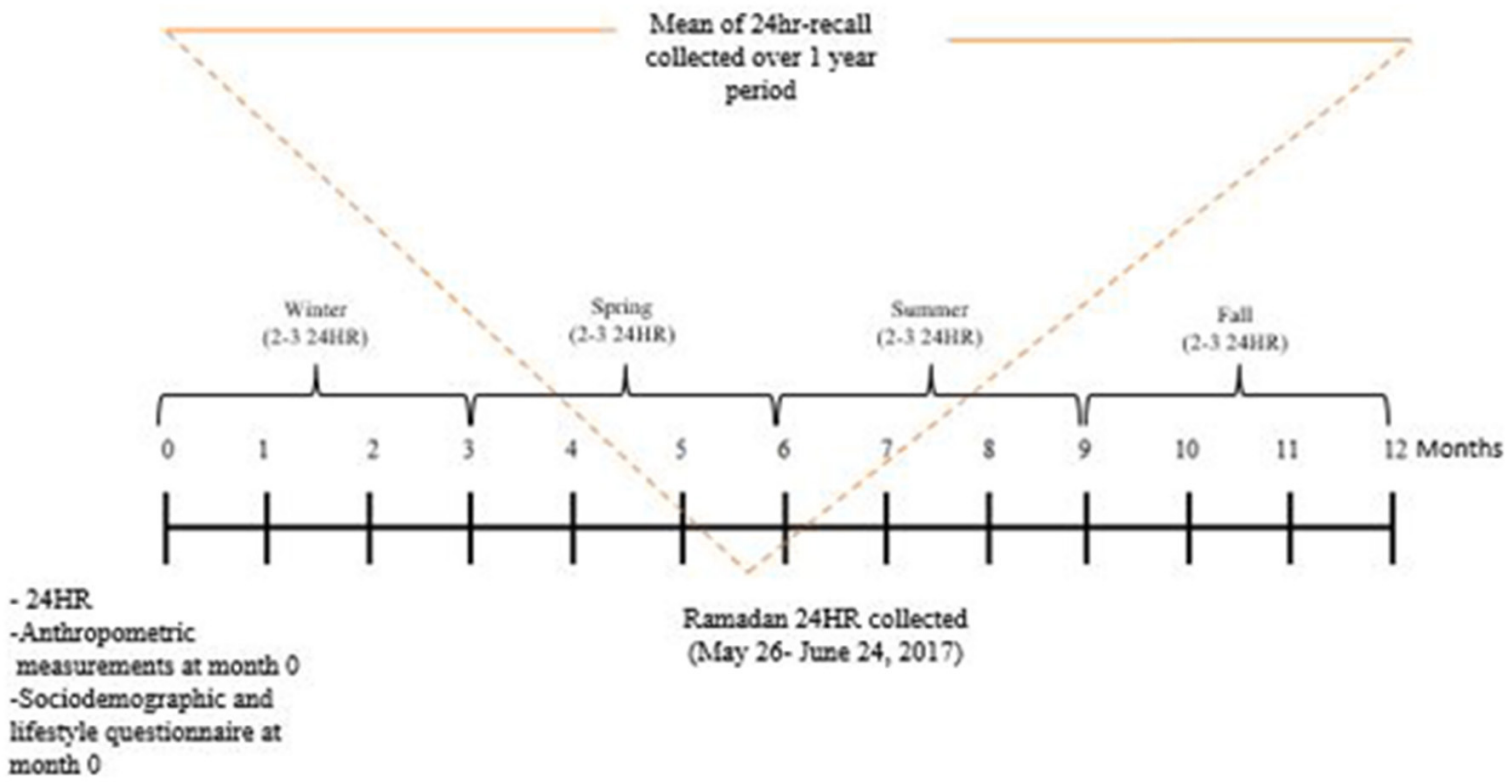
Study protocol.

Every effort was exerted to secure 3 24 HR dietary recalls for each participants during each of the four seasons, totaling to 12 recalls. However, it was inevitable that access to participants be interrupted and only 2 per season were obtained in certain cases. Hence the range in the number of 24 HR dietary recalls. At baseline, a sociodemographic and lifestyle questionnaire was collected with information about the participant's age, sex, place of residence, marital status, smoking, physical activity (Arabic version of the International Physical Activity Questionnaire), education level, type of employment, monthly income, and crowding index (CI). The CI is calculated as the ratio of the number of household residents over the number of rooms in the house and used as a proxy indicator for socioeconomic status in Lebanon and neighboring countries (47–50). Further at the baseline visit, anthropometric measurements of participants were obtained using standardized techniques and calibrated equipment. Weight (kg) was measured to the nearest 0.1 kg using a beam scale. A stadiometer was used to measure the height (cm) to the nearest 0.1 cm. Study subjects were requested to remove their shoes and as much outerwear as possible before measuring the weight and height. The body mass index (BMI) was calculated as weight (kg)/height (m^2^), and classified accordingly into normal (18.5–24.9), overweight (25.0–29.9), and obese (>30 kg/m^2^) (51). All measurements were taken twice and the average of the two readings was used in the analysis. A representation of the study protocol is depicted in Figure 1.

The 24HR was collected using the Multiple Pass Food Recall (MPR) approach that involves a 5- step approach and was developed by the United States Department of Agriculture (USDA) (52, 53). The steps to collect the 24HR using the MPR approach include (1) quick food list recall, (2) forgotten food list probe (3) time and occasion at which foods are consumed, (4) detailed overall cycle, and (5) final probe review of the consumed foods. As a result, for each 24HR collected, the research dietitian obtained information about the time when the meal was consumed, the food consumed, beverages and/or supplement consumed, its portion size, and preparation method. Participants were not aware of the exact day on which the 24HR was collected to maintain the participants' regular dietary habits. The dietitian called the study participant on the same day to schedule the time for the recall. If the participant was not available on the same day (or unreachable), the dietitian would then try to contact the participant on another day. Attempts were repeated until a minimum of 2 24HR recalls were obtained for each season during the 1-year duration of the study. All interviews were administered to each participant by the same-trained research dietitian.

Food items collected from the 24HR were entered into the Nutritionist Pro™ (version 5.1.0, 2014, First Data Bank, Nutritionist Pro, Axxya Systems, San Bruno, CA) and were used to estimate dietary intake of food groups, energy, macro-and micronutrients. Lebanese composite dishes were formulated and added to the software database using standardized recipes based on single food items from the USDA database. All food items from Nutritionist Pro™ were extracted into an excel sheet. To combine food items into groups, a code was given to each food item consumed before entering the data into Statistical Package for Social Sciences (SPSS) (Appendix 1 in Supplementary Material describes the food groups included in the study and their corresponding food items). For each subject, the average calories from each food group consumed were estimated together with their micro-and macronutrients.

### Statistical Analysis

Statistical analysis was computed using Statistical Package for Social Sciences (SPSS, version 25, 2017). The sociodemographic and lifestyle characteristics of the study population were expressed as frequencies and percentages, as well as means and standard deviations (SD) for categorical and continuous variables, respectively. The average intakes (mean ± SD) of the various food groups, energy, macro, and micronutrients were computed for Ramadan and intakes over the remainder of the year. The 24 HR dietary recall collected during Ramadan was excluded from the mean of the dietary intake in regular days. Paired *t*-tests were used to compare dietary intakes between the month of Ramadan and the rest of the year. Food group intakes were expressed as percent contribution to the total energy. To assess the adequacy of selected nutrients, their intake was categorized into those meeting and not meeting recommendations as set by the Institute of Medicine (IOM): the Acceptable Macronutrient Distribution Range (AMDR) for macronutrients and the Dietary Reference Intakes for micronutrients (54). Nutrient adequacy was compared between Ramadan and the rest of the year using the Chi-square test. A *p* < 0.05 was considered statistically significant.

## Results

### Sociodemographic and Lifestyle Characteristics

Table 1 describes the sociodemographic and lifestyle characteristics of study participants (*n* = 62). More than two-thirds (about 67.7%) were <45 years old. Over half of the study participants (56.5%) were residing in Beirut, more than half (59.7%) were married and two-thirds (66.1%) had a university/technical diploma degree. The majority (85.5%) had non-academic employment and 51.6% of the study participants reported a monthly income of 3 million L.L and above. The participants were almost equally distributed between having a crowding index <1 (50%) vs. ≥1 (50%) and being smokers vs. non-smokers (48.4 and 51.6%, respectively). The proportion of normal weight, overweight, and obese participants was 27.4, 40.3, and 32.3%, respectively.

**Table 1:** Sociodemographic and lifestyle characteristics of the study population (*n* = 62).

**Sociodemographic variable**	***N* (%)**
Age (year)	
23–34	25 (40.3)
35–44	17 (27.4)
45 and above	20 (32.3)
Sex	
Male	38 (61.3)
Female	24 (38.7)
Place of residence	
Beirut	35 (56.5)
Outside Beirut	27 (43.5)
Marital status^a^	
Single	25 (40.3)
Married	37 (59.7)
Education level	
Up to high school level	21 (33.9)
University/technical diploma	41 (66.1)
Type of employment	
Academic	9 (14.5)
Non-academic	53 (85.5)
Income per month (L.L.)^b^	
Below 3 million	30 (48.4)
3 million and above	32 (51.6)
Crowding index	
<1	31 (50.0)
≥1	31 (50.0)
Smoking^c^	
Non-smoker	30 (48.4)
Smoker	32 (51.6)
How long have you been a smoker (year) (mean ± SD)	9.54 ± 1.1
Physical activity level	
Low	14 (22.6)
Moderate	39 (62.9)
High	9 (14.5)
BMI (kg/m^2^) (51)	
Normal (18.5–24.9)	17 (27.4)
Overweight (25.0–29.9)	25 (40.3)
Obese (≥30)	20 (32.3)

*BMI, body mass index (kg/m^2^)*.

a *Single including divorced and widowed*,

b *L.L. Lebanese Lira, at the time of data collection 1,500 L.L = 1USD*.

c *Non-smokers including never smoker and past smokers*.

### Comparison of Food Groups' Intakes Between Ramadan and the Rest of the Year

Mean intakes of the food groups, expressed as percent contribution to total energy, for Ramadan and regular days for the study participants are presented in Table 2. While the percent contribution of starchy vegetables and fries, and chips were similar between Ramadan and regular days (*p*-values: 0.410, and 0.311, respectively), that of cereals, cereal-based products, and pasta were significantly higher on regular days (*p* < 0.001). Percent contributions of vegetables and dried fruit intakes to total energy were significantly higher during Ramadan as compared to regular days (vegetables: 13.9 ± 11.3% vs. 8.2 ± 4.7%; *p* < 0.001; dried fruits: 4.8 ± 7.0 vs. 0.5 ± 1.1; *p* < 0.001) while those of fruits, and fruit juices intake were similar (*p* 0.218). Percent contribution of eggs, nuts and seeds, milk and dairy, fats and oils, and olive oil were all higher during regular days in comparison with Ramadan days, with corresponding *p*-values of <0.001, <0.001, 0.017, <0.001, 0.032, and <0.001, respectively. Additionally, chocolate, biscuits, candy, and sugar group and miscellaneous foods intakes (% contribution to total energy) were higher on regular days (*p*-values: 0.005 and 0.002, respectively), whereas intake of Arabic sweets, cakes and pastries, and sugar-sweetened-beverages (% contribution to total energy) were higher in Ramadan with corresponding *p*-values of 0.041 and 0.004, respectively).

**Table 2:** Food groups (percent contribution to energy) during regular days vs. the month of Ramadan among study participants (*n* = 62).

**Food group**	**Regular day intake Mean ± SD**	**Ramadan day intakeMean ± SD**	**Difference between Ramadan and regular days intakes Mean ± SD**	***p*-value^a^**
Cereals, cereal-based products, and pasta	30.5 ± 8.9	18.4 ± 15.2	−12.1 ± 15.5	**<0.001**
Starchy vegetables	0.8 ± 1.1	0.6 ± 1.9	−0.2 ± 2.1	0.410
Fries and chips	5.7 ± 4.6	7.5 ± 13.6	1.8 ± 14.0	0.311
Vegetables and vegetable-based dishes	8.2 ± 4.7	13.9 ± 11.3	5.7 ± 10.9	**<0.001**
Fruits and fresh fruit juice	4.9 ± 3.1	5.9 ± 7.3	0.95 ± 6.9	0.218
Dried fruit	0.5 ± 1.1	4.8 ± 7.0	4.3 ± 7.0	**<0.001**
Meats	7.3 ± 4.7	10.2 ± 13.7	3.0 ± 14.0	0.099
Poultry	5.2 ± 3.9	5.3 ± 8.7	0.1 ± 9.4	0.914
Eggs	1.5 ± 1.7	0.2 ± 0.9	−1.3 ± 2.0	**<0.001**
Fish and seafood	1.4 ± 1.9	1.1 ± 3.8	−0.2 ± 4.1	0.639
Pulses	4.0 ± 3.5	5.2 ± 8.7	1.3 ± 8.5	0.250
Nuts and seeds	2.5 ± 2.9	0.3 ± 1.5	−2.2 ± 3.2	**<0.001**
Milk and dairy products (with yogurt)	6.9 ± 3.8	4.5 ± 7.4	−2.4 ± 7.6	**0.017**
Fats and oils (without olive oil)	2.4 ± 2.2	1.4 ± 3.2	−1.0 ± 3.6	**0.032**
Olive oil	1.7 ± 1.9	0.6 ± 1.7	−1.1 ± 2.2	**<0.001**
Chocolate, biscuits, candies, and sugars (honey and sugar derivatives)	5.1 ± 3.4	2.6 ± 5.9	−2.5 ± 6.7	**0.005**
Arabic sweets, cakes, and pastries	5.9 ± 4.2	8.8 ± 11.7	2.9 ± 10.8	**0.041**
Sugar-sweetened beverages	4.7 ± 3.2	8.4 ± 10.1	3.7 ± 9.7	**0.004**
Miscellaneous	0.7 ± 1.4	0.2 ± 0.9	−0.5 ± 1.1	**0.002**

a *p-value was derived from a paired sample t-test*.

*The numbers in bold are statistically significant (p ≤ 0.05)*.

### Comparison of Energy, Macro- and Micronutrients Intakes Between Ramadan and the Rest of the Year

The mean intakes of energy and nutrients on regular and Ramadan days for study participants are summarized in Table 3. The mean energy intake was slightly but not significantly higher during Ramadan. Similar percent macronutrient contributions to total daily calories were found during Ramadan and regular days and were within the acceptable macronutrient distribution ranges (AMDR) with values around 15% for protein (10–35%), 45% (45–65%) for carbohydrates, and 40% (20–35%) for fats. Cholesterol and saturated fats were significantly higher on regular than Ramadan days (*p*-values: 0.003 and 0.005, respectively). In contrast, sugar and calorie-adjusted dietary fiber intakes were significantly higher in Ramadan (*p*-values: <0.001 and 0.003, respectively). Vitamin A, β-carotene, vitamin C, folate and magnesium were also significantly higher in Ramadan (*p*-values: 0.022, 0.002, 0.014, 0.011 and 0.014, respectively) whereas calcium intake was significantly lower (*p* < 0.001). Further, intakes of sodium, α-carotene, iron, vitamin D, and α-tocopherol were similar between Ramadan and regular days (*p*-values: 0.762, 0.387, 0.180, 0.093, and 0.891, respectively).

**Table 3:** Energy, macro, and micronutrient consumption during regular days vs. the month of Ramadan among study participants (*n* = 62).

**Nutrient**	**Regular day intake Mean ± SD**	**Ramadan day intakeMean ± SD**	**Difference between Ramadan and regular days intakes Mean ± SD**	***p*-value^a^**
Energy (kilocalories)	2040.9 ± 611.2	2184.2 ± 2497.4	143.3 ± 2426.4	0.644
Protein (%)	15.0 ± 2.8	15.2 ± 5.4	0.2 ± 5.7	0.795
Carbohydrates (%)	44.1 ± 5.4	45.9 ± 9.0	1.9 ± 8.8	0.100
Fats, total (%)	40.7 ± 4.6	38.9 ± 7.5	−1.8 ± 8.1	0.078
Cholesterol (mg)	243.5 ± 138.1	175.5 ± 155.7	−68.0 ± 176.1	**0.003**
Saturated fats (%)	10.7 ± 1.9	9.5 ± 3.1	−1.2 ± 3.3	**0.005**
Monounsaturated fats (%)	15.2 ± 3.0	14.3 ± 3.8	−0.9 ± 4.7	0.126
Polyunsaturated fats (%)	9.2 ± 1.8	9.7 ± 3.8	0.5 ± 4.0	0.287
Dietary fibers, total (g)	18.7 ± 6.5	22.2 ± 29.8	3.5 ± 29.9	0.356
Dietary fibers (g/1,000 kcal)	9.3 ± 2.1	11.1 ± 5.2	1.8 ± 4.5	**0.003**
Sugars, total (%)	14.7 ± 4.7	25.5 ± 14.2	10.7 ± 13.7	**<0.001**
Sodium (mg)	2398.1 ± 737.9	2321.2 ± 2090.8	−76.9 ± 1988.8	0.762
Vitamin A (RE)	1041.4 ± 644.6	1697.1 ± 2144.7	655.7 ± 2189.9	**0.022**
Beta-carotene (μg)	3619.9 ± 2341.0	8087.6 ± 10406.8	4467.6 ± 10738.8	**0.002**
Alpha-carotene (μg)	420.7 ± 536.6	893.3 ± 4249.3	472.6 ± 4269.3	0.387
Vitamin C (mg)	84.0 ± 36.8	161.8 ± 237.9	77.7 ± 241.6	**0.014**
Calcium (mg)	806.5 ± 233.0	672.0 ± 264.9	−134.4 ± 255.5	**<0.001**
Iron (mg)	12.9 ± 5.1	14.5 ± 9.5	1.6 ± 9.0	0.180
Vitamin D (μg)	1.3 ± 1.1	0.9 ± 1.9	−0.4 ± 2.0	0.093
Alpha-tocopherol (mg)	10.7 ± 4.0	10.5 ± 13.5	−0.2 ± 14.2	0.891
Folate (total) (μg)	321.7 ± 115.6	413.4 ± 277.9	91.7 ± 275.2	**0.011**
Magnesium (mg)	283.2 ± 90.4	377.4 ± 304.0	94.2 ± 294.0	**0.014**

a *p-value was derived from a paired sample t-test*.

*The numbers in bold are statistically significant (p ≤ 0.05)*.

With regards to nutrient adequacy, the proportions of study participants falling below or above the dietary reference intakes (DRIs) were calculated and compare between regular days and Ramadan (Table 4). Compared to regular days, the proportions of subjects meeting the recommendations for vitamin C, iron and magnesium were significantly higher during Ramadan (53 vs. 43%; 57 vs. 55%; 35 vs. 13%).

**Table 4:** Dietary adequacy during regular days vs. the month of Ramadan among study participants (*n* = 62).

**Nutrient**	DRI^*^	**Regular day intake*N* (%)**	**Ramadan day intake *N* (%)**	***p*-value^§^**
**Dietary fibers, total (g/day)**				
Below recommendation Above recommendation	M:38 F:25	60 (96.8) 2 (3.2)	58 (93.5) 4 (4.5)	0.874
**Sodium (mg/day)**				
Below recommendation Above recommendation	1,500	7 (11.3) 55 (88.7)	23 (37.1) 39 (62.9)	0.244
**Vitamin C (mg/day)**				
Below recommendation Above recommendation	M: 90 F:75	35 (56.5) 27 (43.5)	29 (46.8) 33 (53.2)	**0.017**
**Calcium (mg/day)**				
Below recommendation Above recommendation	1,000	51 (82.3) 11 (17.7)	56 (90.3) 6 (9.7)	0.231
**Iron (mg/day)**				
Below recommendation Above recommendation	M:8 F:18	28 (45.2) 34 (54.8)	27 (43.5) 35 (56.5)	**<0.001**
**Vitamin D (μg/day)**				
Below recommendation Above recommendation	5	61 (98.4) 1 (1.6)	60 (96.8) 2 (3.2)	0.968
**Folate (Total) (μg/day)**				
Below recommendation Above recommendation	400	43 (69.4) 19 (30.6)	44 (71.0) 18 (29.0)	0.132
**Magnesium (mg/day)**				
Below recommendation Above recommendation	M:420 F:320	54 (87.1) 8 (12.9)	40 (64.5) 22 (35.5)	**<0.001**

§ *p-value was derived from the chi-square test*.

*The numbers in bold are statistically significant (p ≤ 0.05)*.

* *DRI for age group 31–50y was used and calculated based on DRI (Institute of Medicine/Food and Nutrition Board. Dietary Reference Intakes for Energy, Carbohydrate, Fiber, Fat, Fatty Acids, Cholesterol, Protein, and Amino Acids.2002/2005). https://www.nap.edu/catalog/10490/dietary-reference-intakes-for-energy-carbohydrate-fiber-fat-fatty-acids-cholesterol-protein-and-amino-acids-macronutrients (54)*.

*M, Males; F, Females*.

**Figure 2:**
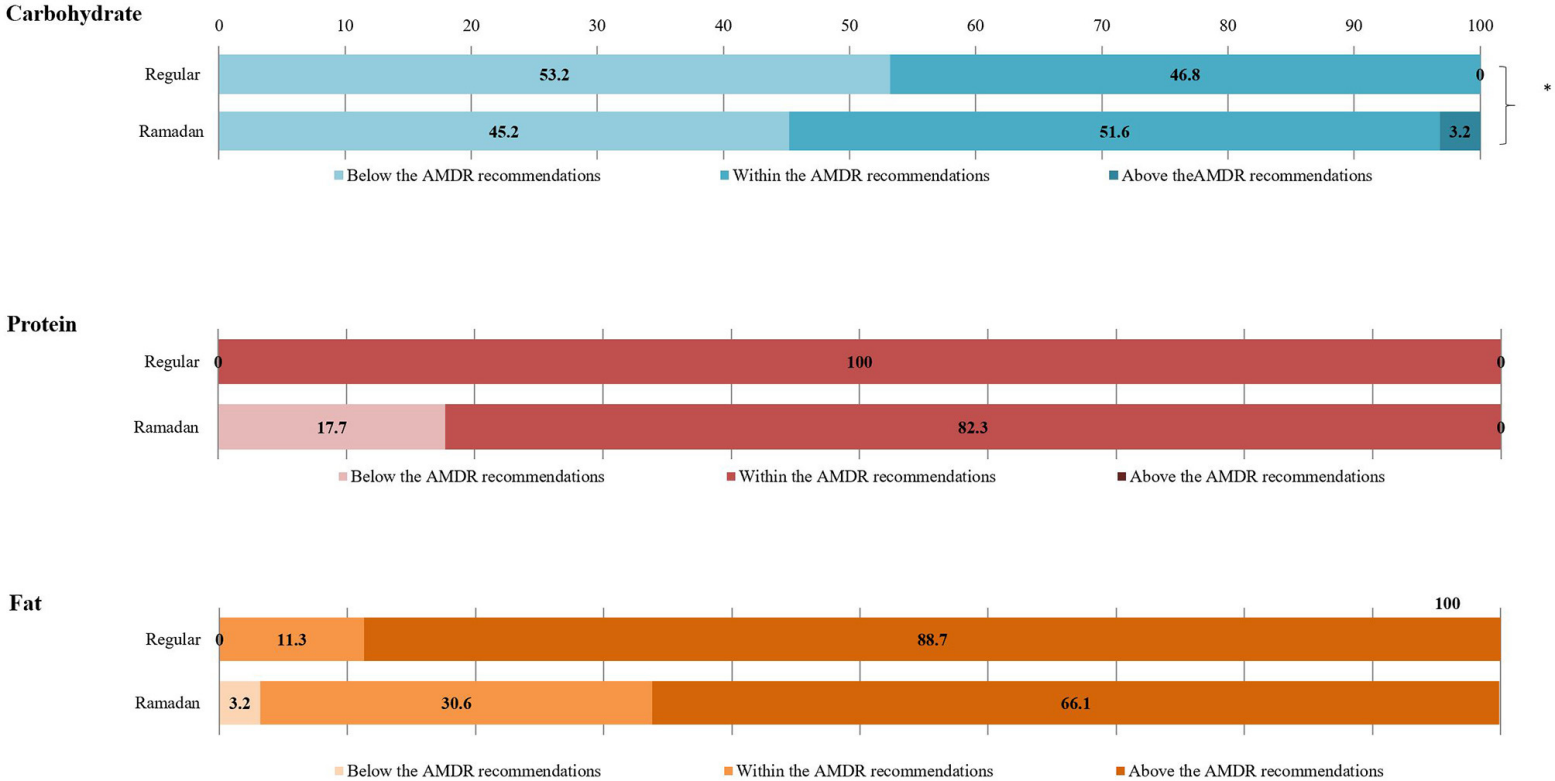
Comparison of the proportions of participants meeting the AMDR recommendations for macronutrients between regular days and the month of Ramadan among study participants (*n* = 62). The ‘*’ denoted a statistically significant difference at 0.05.

Figure 2 depicts the proportion of the study population meeting the AMDRs for daily macronutrients (regular days vs. Ramadan) for the fasting participants. All were within the AMDRs for protein (100.0%) during regular days, compared to 82.3% during Ramadan. Further, 53.2, 46.8, and 0% were below, within, or above AMDRs for carbohydrates during regular days compared to 45.2, 51.6, and 3.2% during Ramadan (*p* < 0.05). Fat intake was above the AMDRs for 88.7% during regular days and 66.1% in Ramadan.

### Gender Differences in the Impact of Ramadan on Dietary Intake

A comparison between dietary intakes of males and females showed that, while a few differences were observed during regular days, their intake was very similar during the month of Ramadan. More specifically, with regards to food groups, women tended to eat more vegetables and vegetable-based dishes and less meat as compared to males during regular days. These differences were not observed during the month of Ramadan (Appendix 2 in Supplementary Material).

### The Impact of Ramadan on Dietary Intake Among Normal vs. Overweight and Obese Study Participants

The impact of Ramadan on dietary intake were analyzed separately or normal and overweight and obese study participants. Results are presented in Appendix 3 (Supplementary Material). Fewer differences in food groups intakes (as expressed in % contribution to total energy intake) were noted among normal as compared to overweight and obese subjects. Similarly, intakes of various nutrients tended to be less different between Ramadan and regular days among normal weight subjects as compared to overweight and obese subjects (Appendix 3 in Supplementary Material).

## Discussion

The current study is the first to examine the dietary intakes during Ramadan and to compare it to that of the rest of the year's months; with the majority of previous research examining dietary intakes before and after Ramadan for 1–2 months at the most (13, 14, 16, 19–26, 55). The year round approach used in this study provides a more precise estimate of the difference between the two time intervals to improve understanding of the impact of religious fasting on dietary behaviors and nutrient intakes. The study showed that Ramadan fasting was associated with significant changes in the intakes of 12 of the 19 food groups, implying that Ramadan has its unique food intake and dietary pattern as compared to the rest of year days. More specifically, percent contribution to energy intake of cereals, cereal-based products, and pasta were decreased, while vegetables and dried fruit intakes were increased during Ramadan. Concomitantly, percent contribution of eggs, nuts and seeds, milk and dairy, fats and oils, and olive oil to total energy were lower during Ramadan. Additionally, intakes of classical confectionaries and sweets such as chocolate, biscuits, candies, and sugar groups and miscellaneous foods (% contribution to total energy) were lower during Ramadan and were replaced by traditional Arabic sweets, cakes, and pastries, and sugar-sweetened-beverages specific to this month of the year.

The observed marked changes in dietary intake in this study further highlight the previously reported profound impact of religious fasting on eating behavior. In fact, religion and religious rituals and fests have for long been considered among the fundamental factors that impact human dietary behaviors and food selections (2). Ramadan fasting represents one of the clearest examples of how religious beliefs impact human dietary behavior for three main reasons: first, the significant shift in food patterns from diurnal and nocturnal eating time to nocturnal eating for 29–30 consecutive days; second, dietary choices during this month are closely tied to traditions and customs with certain dishes consumed solely during Ramadan, and lastly particular eating habits during Ramadan whereby during month, meals are generally consumed together with family members (56, 57).

The results of this study revealed a clear tendency toward increasing intake of sugar-containing foods (dried fruit, Arabic sweets, and sugar sweetened-beverages) during night hours of Ramadan, which mirrors the recent reports from UAE (58, 59), and Ghana (17) indicating increased consumption of sugar-sweetened beverages after breaking the fast, and is in accord with other previous reports from different parts of the world (57, 60, 61). Such a higher consumption of these foods could be linked to common traditions and customs during this month, whereby certain sweets are produced, sold, and consumed solely during the month of Ramadan and not consumed during the rest of year. The marked increase in sugary food intake during this month could be explained by the reported feeling of dizziness and inactivity due to reduced blood sugar during the fasting hours prior to consumption, which drives the fasting individual to consume more sugars between dusk and dawn. This behavior is also closely linked to the traditions of consuming particular sweets that are specific to the month of Ramadan. In this context, it is important to highlight the philosophy of Ramadan fasting which stems from empowering self-control and contradicts with the prophetic guidance dictating avoidance of overeating; as noted in the following: “A human being fills no worse vessel than his stomach. It is sufficient for a human being to eat a few mouthfuls to keep his spine straight. But if he must (fill it), then one-third of food, one third for drink and one third for air” (62), and disagrees with the behaviors of the Messenger of Islam (Prophet Mohamad) who was reported to break his fast with water and few dates, and then taking small meal afterward (63).

Such an increased sugar intake during the month of Ramadan is important to be considered especially among patients with diabetes. While the effect of fasting (and its associated higher sugar intake) did not show any adverse glucometabolic impacts in healthy subjects (64), its effects on diabetic patients remains to be elucidated. Worldwide, it is estimated that 70 million people with diabetes observe fasting during Ramadan (65). Considering this large population of patients who mostly self-decide to fast during Ramadan, despite being exempted to do so as per the Islamic rules of Ramadan, the findings of this study (higher sugar consumption during the month of Ramadan) ought to be integrated into the formulation of culture-specific recommendations for dietary intake during the month of Ramadan for this group of patients. Furthermore, health care providers are encouraged to follow more closely the glycemic control of their diabetic patients during the month of Ramadan.

The current study showed a significant reduction in cereals and cereal-based starchy foods and pasta during the month of Ramadan compared to the rest of the year, which is consistent with the reported reduction in consuming breads and cereals in Iran by Nachvak et al. (26). While a slight, non-significant increase in protein sources of food (meats, poultry, and pulses) was reported in the current work, other reports indicated a significant increase in the consumption of protein foods during Ramadan (16, 23, 25, 27). It is arguable that the reported higher intakes of proteins during the month of Ramadan is related to its satiating effect. In fact, the results of a meta-analysis examining the effects of increased protein intake on fullness showed that higher protein meals increase fullness ratings more than lower protein meals (66). The increased intake of non-starchy vegetables and vegetable-based dishes reported in the current study in Lebanon echoes the reported increased consumption of vegetables in the United Arab of Emirates (UAE) during the holy month of Ramadan (14). In this study, the consumption of vegetables and dried fruit intakes was higher during Ramadan compared to the rest of the year. In fact, during this month, salads, dates, and dried apricots constitute an important cultural and traditional components of the meals (67) and hence their observed higher consumption in this study.

Studies examining the effect of Ramadan fasting on fat intake yielded inconsistent results. The findings of this study, together with another study from Spain (23), showed lower intake of fats and oils especially saturated fats. These findings are in line with the findings of Adlouni et al. who showed that fasting during Ramadan induces a marked increase in high-density lipoprotein cholesterol and decrease in low-density lipoprotein cholesterol (16). No such findings were reported in a study from the UAE which reported higher intakes of fats during Ramadan (14). Such inconsistency could be a reflection of the cultural context where the studies have been conducted.

In this study, the increased intake of dietary fibers, vitamin C, beta-carotene, and vitamin A (expressed as retinol equivalent) during Ramadan compared to the non-fasting remainder of the year could be explained by a higher intake of vegetables, and dried fruit during Ramadan night hours. This increased intake of fiber-rich foods could be looked at as a prophylactic measure for the anticipated and frequently experienced constipation during the fasting hours, which is exaggerated with the lack of water intake and reduced physical activity during Ramadan (68). Besides, fresh and dried fruits and vegetable salads are principal ingredients in many dishes during and out of Ramadan in the traditional Lebanese cuisine, which is part of the Mediterranean diet pattern (42).

The lack of significant change in total caloric intake reported in the current work is in line with the findings of many studies in different parts of the world (23, 58, 69–72). Accordingly, a few previous studies proposed Ramadan as a variant of intermittent fasting (73). In fact, the comparable total energy and macronutrient intakes during Ramadan and regular days in this study represents one of the distinguishing features of an intermittent fasting regimen (74). Having dietary intakes of macronutrients within the acceptable reference range for protein, carbohydrate, and fat, with higher intakes of many of the micronutrients (vitamin A, β-carotene, vitamin C, folate, and magnesium) and fiber, suggests that an intermittent fasting regimen, such as the one followed in Ramadan, may be a more practical dietary modification than caloric restriction (75).

Of note are the findings of this study showing that, while dietary intake of men and women tend to differ during the year, it is less different during the month of Ramadan. This finding could be due to the fact that during this month, families (including men and women) tend to eat together more frequently as compared to the rest of the year. The analysis by weight status in this study showed that more changes in dietary intake during Ramadan were observed among overweight and obese subjects as compared to those with normal weight. This finding is in line with the results reported by Fernando et al. and which showed a reduction of fat percentage in overweight and obese people during Ramadan fasting but not normal weight (76). Future larger studies are needed to further elucidated the differential effect of Ramadan on dietary intake among normal, overweight, and obese individuals.

The differences among various studies' findings regarding the effect of the month of Ramadan on dietary intake could relate to methodologic and cultural differences in study populations. One leading factor for discrepant results across studies examining changes in food and dietary intakes is the variation in dietary assessment tools. The latter being tightly related to the objective of the dietary assessment; whether assessing general patterns or explicit nutrient intakes for correlational purposes. More specifically, studies that aimed at providing a broader reflection of the dietary pattern generally tended to use food frequency questionnaires (FFQ); while those evaluating particular nutrients reported using repeated dietary recalls and records (58, 59, 77). In addition to the differences in their application, the use of various dietary assessment methods is bound to feasibility and budgetary issues. For instance, FFQ is a relatively inexpensive option and requires less commitment from the participants as compared to dietary recalls and records (78, 79). Therefore, caution ought to be exerted when comparing the results of various studies with different dietary assessment methods. Another major factor for the discrepancies among the studies addressing Ramadan eating is the unique cultural and traditional dietary behaviors adopted by different populations from various ethnic and cultural backgrounds, all within the regulations of the allowed *Halal* food system for Muslims.

To the best of our knowledge, this is the first study to examine the impact of Ramadan fasting on dietary intakes among healthy adults using a year round dietary assessment. As such, the results of this study were more reflective of the subjects' usual dietary habits around the year while those of other studies, which assessed the dietary intake 1 or 2 months before or after Ramadan, tend to be less representative to the overall dietary pattern of the individual. That said, the findings of this study ought to be interpreted in light of a few limitations. Dietary assessment was carried out using multiple 24 HR. Though this method is easy and simple, it relies on the memory of the participants, and hence results could be subject to recall error and bias. For that, project research assistants were trained to use the multiple pass technique to decrease such errors. Furthermore, the fact that only one of the 24 HR was carried out during the month of Ramadan may raise questions with regards to its representation of dietary intake during this month. That said, it is arguable that food intake during the fasting month tend to follow a consistent pattern especially with regards to the type and quantity of food intake. In addition, some of the changes observed in this study could be due to the time of the year Ramadan coincided with; perhaps future studies during years where Ramadan falls in different seasons will help decipher food habit changes due to Ramadan vs. those due to seasonal variations. Whether the observed impact of fasting on dietary intake is more pronounced during the months around Ramadan and fades through the year or such an impact is rather sustained for longer periods remains to be elucidated. Lastly, despite the advantages of an interview-based data collection, the possibility of an interviewer bias or a social desirable response could not be ruled out. Interviewers, however, were trained to maintain non-judgmental and neutral attitudes to avoid impacting the participants' responses.

## Conclusions

In conclusion, the findings of this study suggested major changes in dietary intakes of Lebanese adults observant of the fast during the month of Ramadan as compared to the rest of the year. Such changes included a decrease in the usual intakes of cereals, cereal-based products, pasta, eggs, nuts and seeds, milk and dairy, fats and oils, and olive oil. On the other hand, fasting Lebanese adults were shown to consume more vegetables, dried fruit intakes, traditional Arabic sweets, cakes and pastries, and sugar-sweetened-beverages specific to the month of Ramadan. With the large number of adults who observe fasting during Ramadan, the particularities of dietary intake during Ramadan ought to be considered in the formulation of context and culture-specific dietary recommendations. To overcome the limitations of this study, future studies addressing the changes in dietary habits in Ramadan are encouraged to include more than one daily dietary intake collection and to further characterize dietary intake by eating occasions. Furthermore, given the subjectivity of most dietary assessment methods, if feasible, it will be optimal to collect biomarkers of various nutrients intake to compare between Ramadan and the rest of the year (80).

## Data Availability Statement

The raw data supporting the conclusions of this article will be made available by the authors, without undue reservation.

## Ethics Statement

The studies involving human participants were reviewed and approved by Institutional Review Board of the Social and Behavioral Sciences at the AUB. The patients/participants provided their written informed consent to participate in this study.

## Author Contributions

FN, MFo, and MFa contributed to the conception and design of the research. HS contributed to the study management. RE, MB, and HS contributed to data collection and preparation of the project data set. HS and NA undertook the analysis. FN, MFa, RC, and HS contributed to drafting the manuscript. All authors reviewed the manuscript and approved the final version intended for submission.

## Conflict of Interest

The authors declare that the research was conducted in the absence of any commercial or financial relationships that could be construed as a potential conflict of interest.

## Publisher's Note

All claims expressed in this article are solely those of the authors and do not necessarily represent those of their affiliated organizations, or those of the publisher, the editors and the reviewers. Any product that may be evaluated in this article, or claim that may be made by its manufacturer, is not guaranteed or endorsed by the publisher.
