# Supplementary material for: Impact of Ramadan Fasting on Dietary Intakes Among Healthy Adults: A Year-Round Comparative Study
Source: Front Nutr. 2021 Aug 5;8:689788. doi: 10.3389/fnut.2021.689788 (PMC8375294; doi:10.3389/fnut.2021.689788)
Supplement: Supplementary file 1 [file Table_1.docx]

Supplementary Material

**Appendix 1.** List of food items in each food group in Table 4.

| **Group number** | **Food groups** | **Food items obtained from the FFQ and the 24 HR** |
| --- | --- | --- |
| 1 | Cereals, cereal-based products, and pasta | Pita bread white, whole wheat pita bread, Arabic flatbread, tannour, brioche, French toast, French toast whole wheat, bagels, white French baguette, whole French baguette, oat bran bread, toasted rye bread, cereal bar, cereal granola, kaak (finger or round), bran or whole-grain breakfast cereals, regular cereals, puffed corn chocolate flavored, toast and crackers, muesli, dried fruit and nuts cereal, oats, dry, hard salted pretzel, hamburger bun, flour (white/ coconut), garlic bread. Chicken with rice, moghrabieh (pearl couscous with meat and chicken), pumpkin kibbe, lasagna, macaroni gratin, pasta, pasta with yogurt and garlic, rice with vermicelli, spaghetti, tortellini, mudardara (lentil and rice), couscous, composite pasta cooked, plain noodles, white rice, brown rice, fried rice, rice with meats and nuts, Ground Psyllium Seeds  Bulgur cooked, bulgur with vermicelli, bulgur with tomato, bulgur with chicken/meat), freekeh cooked quinoa. Manaeesh (cheese, zaatar, kishk), manouche made with flatbread (cheese, zaatar), pizza (vegetarian, pepperoni, margarita,  pies, spinach fatayer, sambousak meat/ cheese, fried cheese rolls, meat pie, Lebanese meat dumplings |
| 2 | Starchy vegetables | Potato, oven-baked potato (with or without beef), spicy fried potato cubes with garlic and coriander, stuffed potato with meat, kishk with potato, potato kebbe, potato stew with meat, potato, and squash soup, baked/ boiled potato, mashed potato, nachos, potato wedges, sweet Russian salad, tortilla corn, canned corn |
| 3 | Fries and chips | French fries, potato chips regular, potato chips light, |
| 4 | Vegetables and vegetable-based dishes | Canned asparagus, canned carrot juice, canned mushroom/peas, frozen vegetable mix. Carrot juice, green salad, seasonal salad, dark green leafy vegetables, carrots, tomatoes, cucumber, spinach, thyme, beetroot, pepper, green chicory, lettuce/cabbage/broccoli/kale, Mint leaves, parsley, radishes, fresh thyme, arugula, chopped onions, garlic clove, ginger, lemon juice, rocket and green thyme salad, tabbouleh tomato grilled with garlic, Caesar salad, boiled mushroom, tomato sauce canned, beetroot salad, fattoush, coleslaw (with mayonnaise/ with lemon garlic), boiled broccoli/cauliflower, vegetable soup, chicory fried with onions, zucchini with oil, oven-baked vegetable mix, eggplant, zucchini, cabbage, chard beet leaves, grape leaves (stuffed with rice and meat), green bean stew with meat, molokhiya, okra/peas stew with meat, spinach, artichoke, eggplant, cauliflower cooked. |
| 5 | Fruits and fresh fruit juice | Loquats, tangerines, apple, custard (Bullock's Heart or Cherimoya), apricots, fruits salad, blackberries, blueberries, cantaloupe, guava, cherries, strawberries, cranberries, apples, bananas, oranges, kiwi, mango, pears, plums, persimmon, pineapple, raspberries, peaches, pomegranate, figs, watermelon, grapes, fruit-based desserts (cocktails), canned fruits (pineapple, plums) fresh orange juice, fresh grapefruit juice |
| 6 | Dried fruit | Dried fruits (dates, apricots, raisins, figs). |
| 7 | Meats | Cured meat: Ham, mortadella (beef/pork), hotdog, turkey, minced meat, sausages, kibbe, hreese with meat, vegetable stew with meat (mixed dishes), kafta (ground beef mixed with herbs), shawarma meat, lamb, Steak beef/veal, kafta with potato in the oven, meat-based kibbe in yogurt, meat fajita, sandwich beef shawarma, soup rice with meatballs, beef stroganoff, meat (lamb/beef): low medium high fat/ cooked, luncheon meats, hamburger. Offal: Organ meats, beef brain (and organ meat), liver (chicken, lamb), heart (raw, pan-fried) |
| 8 | Poultry | Poultry (breast, thigh, leg, wing, drumstick), nuggets, escalope, chicken curry, chicken fajita, Chinese chicken, sandwich chicken shawarma, shawarma chicken, tawook (grilled chicken skewers), chicken crispy/tenders/fingers, chicken patty, quesadilla chicken |
| 9 | Eggs | Oriental omelet, eggs with potato, tomatoes, and eggs, whole eggs, egg whites, fried/boiled eggs, |
| 10 | Fish and seafood | Canned fish with oil, canned fish in water, fresh/frozen fish, whole/ fillet fish, salmon, sardine, shellfish (shrimp, calamari, crab), sushi, |
| 11 | Pulses | Beans, chickpeas/lentils/ fava beans, falafel, red beans stew with meat, red beans in oil, fatteh hummus (chickpeas in yogurt and fried bread), fava beans with bulgur, fava beans with chard, mujaddara (lentil and rice), soup lentil, lentil salad, canned beans |
| 12 | Nuts, and seeds | Green almond, shredded coconut, raw nuts and seeds, roasted and unsalted nuts and seeds, roasted and salted nuts and seeds, peanuts, peanut butter, edamame, mixed nuts, seeds, soybeans |
| 13 | Milk and dairy products  (with yogurt) | Cheese (low fat/ light/ white), double cream cheese, feta, shaklish (herbed cheese balls), halloumi, akkawi, cheese (high fat/yellow), kashkaval, brie, goat, gouda, mozzarella, swiss, cheddar, parmesan, cheese (processed/ creamy), spinach four cheese, mozzarella sticks,  lactose-free milk, dry milk (dry, non-fat), milkshake, full-fat milk, low fat, fat-free milk, hot chocolate mix prepared with 2 % milk, hot chocolate, cream fraiche, kashta (clotted cream), whipped cream. Labneh (regular, low fat, skim), yogurt (regular, light), cooked yogurt, salty yogurt drink, Greek yogurt (whole milk, low fat), laban immo (yogurt with meat stew) |
| 14 | Fats and oil | Butter/ghee, mayonnaise (regular, light), tahini, vegetable oil, vegetable ghee, guacamole sauce, hummus with tahini, tarator, sesame oil, coconut oil, flaxseed oil, avocado |
| 15 | Olive oil | Olive oil, olives (green, black) |
| 16 | Chocolate, biscuits, candy, and sugar  (honey and sugar derivatives) | Sugar (white, brown), candy, jellybeans, marshmallow, chocolate (milk, dark), chocolate spread, chocolate syrup, cookie, wafer, Jam, molasses, halawa, honey, maple syrup, ice cream (regular, low fat),  Jello, regular pudding (custard, milk pudding, milk pudding with rice, mghli (spiced rice pudding), snayneye (shelled wheat pudding), low-fat pudding. |
| 17 | Arabic sweets, cakes, and pastries | Cakes and pastries, unfrosted muffins, frosted muffins, plain biscuits, stuffed biscuits, wafer biscuits croissant, doughnuts, Kaak with milk, cookies, crepes, pancakes, delights  Arabic sweets. |
| 18 | Sugar-sweetened beverages | Instant coffee powder/prepared, cappuccino, Turkish coffee, 2 in 1, 3 in 1 Nescafe, coffee creamer (original, fat-free), tea, decaffeinated coffee, iced tea, herbal tea, energy and sport drinks, soda regular, soda diet, water, sparkling water, canned fruit juices: kiwi strawberry drink, drink mix orange flavor prepared with water (Tang^TM^), tamarind, canned fruit juices, jallab, aloe vera, lemonade, mulberry juice, apple/pineapple/grape/ cranberry juice |
| 19 | Miscellaneous | Mustard, zaatar, ketchup, pickles (cucumber, eggplant) |

(U.S. Department of Agriculture and U.S. Department of Health and Human Services, 2010); (American Diabetes Association and American Dietetic Association, n.d.); (Hwalla, N.., Nasreddine, L. & Farhat Jarrar, S., 2012)
