# Supplementary material for: Impact of Ramadan Fasting on Dietary Intakes Among Healthy Adults: A Year-Round Comparative Study
Source: Front Nutr. 2021 Aug 5;8:689788. doi: 10.3389/fnut.2021.689788 (PMC8375294; doi:10.3389/fnut.2021.689788)
Supplement: Supplementary file 2 [file Table_2.DOCX]

Supplementary Material

**Appendix 2. Percent contribution of food groups to total energy* by sex during regular days and during Ramadan days (n=62)**

| **Food groups** | ***Regular days*** | | | ***Ramadan*** | | |
| --- | --- | --- | --- | --- | --- | --- |
|  | **Male**  **Mean ± SD**  **n=38** | **Female**  **Mean ± SD**  **n=24** | ***P*-value ^B^** | **Male**  **Mean ± SD**  **n=38** | **Female**  **Mean ± SD**  **n=24** | ***P*-value ^B^** |
| Cereals, cereal-based products, and pasta | 32.2±9.5 | 27.7±7.3 | 0.053 | 17.2±15.8 | 20.3±14.2 | 0.449 |
| Starchy vegetables | 0.8±1.1 | 0.9±1.0 | 0.804 | 0.6±2.1 | 0.5±1.7 | 0.796 |
| Fries and chips | 5.9±4.3 | 5.3±5.2 | 0.597 | 7.2±9.6 | 7.9±18.5 | 0.854 |
| Vegetables and vegetable-based dishes | 6.2±3.1 | 11.4±5.2 | **0.000** | 12.4±11.4 | 16.2±10.9 | 0.200 |
| Fruits and fresh fruit juice | 4.8±3.0 | 5.2±3.4 | 0.620 | 5.8±8.1 | 6.0±6.1 | 0.904 |
| Dried fruit | 0.5±0.8 | 0.6±1.5 | 0.573 | 5.6±7.5 | 3.6±6.1 | 0.285 |
| Meats | 8.1±5.2 | 5.9±3.4 | **0.044** | 12.2±13.2 | 7.2±14.4 | 0.161 |
| Poultry | 5.5±4.1 | 4.7±3.5 | 0.458 | 4.3±8.0 | 6.9±9.6 | 0.250 |
| Eggs | 1.7±1.9 | 1.1±1.5 | 0.199 | 0.1±0.9 | 0.2±0.9 | 0.874 |
| Fish and seafood | 0.9±1.2 | 2.1±2.4 | **0.009** | 1.0±3.6 | 1.4±4.1 | 0.670 |
| Pulses | 3.9±3.6 | 4.1±3.4 | 0.759 | 5.1±9.5 | 5.4±7.4 | 0.918 |
| Nuts and seeds | 2.3±2.3 | 2.9±3.8 | 0.488 | 0.3±1.3 | 0.4±1.8 | 0.867 |
| Milk and dairy products (with yogurt) | 6.8±3.5 | 7.1±4.4 | 0.792 | 4.4±8.3 | 4.7±5.8 | 0.892 |
| Fats and oils (without olive oil) | 2.5±2.5 | 2.2±1.5 | 0.600 | 1.5±3.1 | 1.2±3.4 | 0.797 |
| Olive oil | 2.0±2.0 | 1.2±1.5 | 0.076 | 0.5±1.4 | 0.7±2.1 | 0.727 |
| Chocolate, biscuits, candies, and sugars  (honey and sugar derivatives) | 5.1±2.9 | 5.2±4.3 | 0.913 | 2.9±6.9 | 2.2±3.8 | 0.626 |
| Arabic sweets, cakes, and pastries | 5.5±4.1 | 6.6±4.5 | 0.318 | 9.4±11.9 | 7.8±11.6 | 0.600 |
| Sugar-sweetened beverages | 4.4±3.1 | 5.1±3.3 | 0.442 | 9.2±10.5 | 7.1±9.3 | 0.428 |
| Miscellaneous | 0.6±0.9 | 0.8±1.9 | 0.528 | 0.1±0.3 | 0.4±1.5 | 0.138 |

^B^ *p*-value was derived from independent sample t- test

The numbers in bold are statistically significant (*p*-value ≤ 0.05).
