# Supplementary material for: Impact of Ramadan Fasting on Dietary Intakes Among Healthy Adults: A Year-Round Comparative Study
Source: Front Nutr. 2021 Aug 5;8:689788. doi: 10.3389/fnut.2021.689788 (PMC8375294; doi:10.3389/fnut.2021.689788)
Supplement: Supplementary file 3 [file Table_3.DOCX]

**Table 2a. Food groups (percent contribution to energy) during regular days vs. the month of Ramadan among study participants with normal weight (n=17).**

| Food group | Regular day intake  Mean ± SD | Ramadan day intake  Mean ± SD | Difference between Ramadan and regular days intakes  Mean ± SD | *p*-value ^a^ |
| --- | --- | --- | --- | --- |
| Cereals, cereal-based products, and pasta | 29.6±8.8 | 22.3±15.5 | -7.3±16.2 | 0.082 |
| Starchy vegetables | 0.7±1.1 | 0.7±2.0 | -0.04±2.9 | 0.951 |
| Fries and chips | 5.1±5.0 | 2.1±4.9 | -3.0±6.0 | 0.057 |
| Vegetables and vegetable-based dishes | 10.1±6.1 | 13.4±8.0 | 3.3±9.7 | 0.174 |
| Fruits and fresh fruit juice | 5.3±3.2 | 3.8±3.6 | -1.5±3.9 | 0.138 |
| Dried fruit | 0.8±1.8 | 3.3±5.4 | 2.5±5.1 | 0.060 |
| Meats | 7.3±4.7 | 10.2±13.7 | 2.0±13.9 | 0557 |
| Poultry | 4.4±4.2 | 5.9±7.4 | 1.5±9.5 | 0.527 |
| Eggs | 1.3±1.5 | 0.3±1.0 | -1.0±1.9 | **0.043** |
| Fish and seafood | 1.3±1.6 | 2.0±4.8 | 0.6±4.3 | 0.564 |
| Pulses | 4.4±3.8 | 6.1±7.4 | 1.7±7.3 | 0.344 |
| Nuts and seeds | 2.6±3.9 | 0.5±2.1 | -2.1±4.2 | 0.054 |
| Milk and dairy products (with yogurt) | 7.9±4.8 | 6.6±6.1 | -1.3±6.8 | 0.438 |
| Fats and oils (without olive oil) | 2.1±1.6 | 1.8±3.9 | -0.3±4.5 | 0.780 |
| Olive oil | 1.5±1.8 | 0.5±2.1 | -1.0±2.3 | 0.096 |
| Chocolate, biscuits, candies, and sugars  (honey and sugar derivatives) | 5.2±2.7 | 2.7±4.3 | -2.5±5.2 | 0.066 |
| Arabic sweets, cakes, and pastries | 6.2±4.2 | 10.9±13.0 | 4.7±11.6 | 0.114 |
| Sugar-sweetened beverages | 4.7±2.7 | 8.8±7.2 | 4.1±7.0 | **0.029** |
| Miscellaneous | 0.9±2.1 | 0.5±1.7 | -0.4±1.1 | 0.157 |

^a^ *p*-value was derived from a paired sample t-test.

The numbers in bold are statistically significant (*p*-value ≤ 0.05).

**Table 3a. Energy, macro, and micronutrient consumption during regular days vs. the month of Ramadan among study participants with normal weight (n=17).**

| **Nutrient** | **Regular day intake**  **Mean ± SD** | **Ramadan day intake**  **Mean ± SD** | **Difference between Ramadan and regular days intakes**  **Mean ± SD** | ***p*-value ^a^** |
| --- | --- | --- | --- | --- |
| Energy (Kilocalories) | 1881.1±421.8 | 1946.8±802.3 | 65.7±517.3 | 0.607 |
| Protein (%) | 14.9±3.1 | 17.0±4.8 | 2.1±5.2 | 0.124 |
| Carbohydrates (%) | 43.2±4.0 | 43.6±8.8 | 0.4±8.5 | 0.834 |
| Fats, Total (%) | 41.9±3.5 | 39.5±6.7 | -2.5±7.5 | 0.193 |
| Cholesterol (mg) | 214.5±134.5 | 186.7±97.7 | -27.8±127.5 | 0.382 |
| Saturated Fats (%) | 11.5±1.6 | 9.8±2.7 | -1.6±3.0 | **0.039** |
| Monounsaturated Fats (%) | 15.5±2.9 | 13.1±3.5 | -2.4±3.9 | **0.021** |
| Polyunsaturated Fats (%) | 9.1±1.7 | 10.0±4.2 | 0.9±4.5 | 0.433 |
| Dietary Fibers, Total (g) | 17.2±4.5 | 18.1±6.5 | 0.9±6.1 | 0.568 |
| Dietary Fibers, (g/1000kcal) | 9.4±2.5 | 9.9±3.7 | 0.5±3.3 | 0.504 |
| Sugars, Total (%) | 15.3±3.9 | 22.3±11.5 | 7.0±10.7 | **0.016** |
| Sodium (mg) | 2227.1±553.3 | 2223.7±1049.8 | -3.5±954.4 | 0.988 |
| Vitamin A (RE) | 999.2±630.4 | 2057.5±3549.4 | 1058.3±3500.5 | 0.231 |
| Beta-Carotene (μg) | 3855.2±2524.7 | 9660.2±17329.1 | 5805.0±17985.6 | 0.202 |
| Alpha-Carotene (μg) | 378.5±241.2 | 1959.7±7588.4 | 1581.2±7568.9 | 0.387 |
| Vitamin C (mg) | 83.3±40.4 | 199.4±303.2 | 116.2±303.2 | 0.134 |
| Calcium (mg) | 839.1±149.2 | 802.7±241.8 | -36.3±282.1 | 0.603 |
| Iron (mg) | 11.9±3.8 | 15.0±7.6 | 3.2±5.7 | **0.036** |
| Vitamin D (μg) | 1.2±1.1 | 0.9±1.6 | -0.3±1.8 | 0.549 |
| Alpha-Tocopherol (mg) | 10.2±2.9 | 9.2±4.6 | -0.9±5.0 | 0.453 |
| Folate (Total) (μg) | 299.8±89.6 | 390.6±159.3 | 90.8±167.9 | **0.040** |
| Magnesium (mg) | 266.4±81.6 | 359.9±133.8 | 93.5±109.4 | **0.003** |

^a^ *p*-value was derived from a paired sample t-test.

The numbers in bold are statistically significant (*p*-value ≤ 0.05).

**Table 2b. Food groups (percent contribution to energy) during regular days vs. the month of Ramadan among Overweight/Obese study participants (n=45).**

| Food group | Regular day intake  Mean ± SD | Ramadan day intake  Mean ± SD | Difference between Ramadan and regular days intakes  Mean ± SD | *p*-value ^a^ |
| --- | --- | --- | --- | --- |
| Cereals, cereal-based products, and pasta | 30.8±8.7 | 17.0±14.9 | -13.9±15.0 | **<0.001** |
| Starchy vegetables | 0.8±1.1 | 0.5±1.9 | -0.3±2.1 | 0.348 |
| Fries and chips | 5.9±4.5 | 9.5±15.2 | 3.6±15.7 | 0.128 |
| Vegetables and vegetable-based dishes | 7.5±4.0 | 14.1±12.3 | 6.5±11.3 | **<0.001** |
| Fruits and fresh fruit juice | 4.8±3.1 | 6.7±8.2 | 1.9±7.5 | 0.103 |
| Dried fruit | 0.4±0.7 | 5.4±7.5 | 4.9±7.6 | **<0.001** |
| Meats | 7.8±4.8 | 11.2±14.0 | 3.4±14.2 | 0.112 |
| Poultry | 5.5±3.8 | 5.1±9.2 | -0.4±9.4 | 0.785 |
| Eggs | 1.5±1.8 | 0.1±0.8 | -1.4±1.9 | **<0.001** |
| Fish and seafood | 1.4±1.9 | 0.8±3.3 | -0.6±4.0 | 0.342 |
| Pulses | 3.8±3.4 | 4.9±9.1 | 1.1±9.0 | 0.426 |
| Nuts and seeds | 2.5±2.5 | 0.3±1.2 | -2.2±2.7 | **<0.001** |
| Milk and dairy products (with yogurt) | 6.5±3.4 | 3.8±7.7 | -2.8±8.0 | **0.024** |
| Fats and oils (without olive oil) | 2.5±2.4 | 1.2±2.9 | -1.3±3.2 | **0.011** |
| Olive oil | 1.7±1.9 | 0.6±1.5 | -1.2±2.2 | **0.001** |
| Chocolate, biscuits, candies, and sugars  (honey and sugar derivatives) | 5.1±3.7 | 2.6±6.4 | -2.5±7.3 | **0.027** |
| Arabic sweets, cakes, and pastries | 5.8±4.3 | 8.0±11.3 | 2.2±10.6 | 0.173 |
| Sugar-sweetened beverages | 4.7±3.3 | 8.3±11.0 | 3.6±10.6 | **0.028** |
| Miscellaneous | 0.6±1.0 | 0.1±0.4 | -0.5±1.1 | **0.005** |

^a^ *p*-value was derived from a paired sample t-test.

The numbers in bold are statistically significant (*p*-value ≤ 0.05).

**Table 3b. Energy, macro, and micronutrient consumption during regular days vs. the month of Ramadan among Overweight/Obese study participants (n=45).**

| **Nutrient** | **Regular day intake**  **Mean ± SD** | **Ramadan day intake**  **Mean ± SD** | **Difference between Ramadan and regular days intakes**  **Mean ± SD** | ***p*-value ^a^** |
| --- | --- | --- | --- | --- |
| Energy (Kilocalories) | 2101.2±663.0 | 2273.8±2895.3 | 172.6±2839.3 | 0.685 |
| Protein (%) | 15.1±2.7 | 14.5±5.6 | -0.5±5.7 | 0.544 |
| Carbohydrates (%) | 44.4±5.9 | 46.8±9.0 | 2.4±8.9 | 0.077 |
| Fats, Total (%) | 40.3±5.0 | 38.7±7.8 | -1.6±8.4 | 0.206 |
| Cholesterol (mg) | 254.5±139.3 | 171.3±173.4 | -83.2±190.3 | **0.005** |
| Saturated Fats (%) | 10.4±1.9 | 9.4±3.3 | -1.0±3.4 | **0.047** |
| Monounsaturated Fats (%) | 15.1±3.1 | 14.7±3.8 | -0.4±4.9 | 0.623 |
| Polyunsaturated Fats (%) | 9.2±1.9 | 9.6±3.8 | 0.4±3.9 | 0.467 |
| Dietary Fibers, Total (g) | 19.2±7.1 | 23.8±34.6 | 4.5±35.0 | 0.389 |
| Dietary Fibers, (g/1000kcal) | 9.2±2.0 | 11.5±5.6 | 2.2±4.8 | **0.003** |
| Sugars, Total (%) | 14.5±5.0 | 26.6±15.0 | 12.1±14.5 | **<0.001** |
| Sodium (mg) | 2462.7±792.5 | 2358.0±2377.9 | -104.7±2269.3 | 0.758 |
| Vitamin A (RE) | 1057.4±656.2 | 1561.0±1314.1 | 503.6±1451.4 | **0.025** |
| Beta-Carotene (μg) | 3531.1±2291.4 | 7493.5±6295.3 | 3962.4±6426.2 | **<0.001** |
| Alpha-Carotene (μg) | 436.6±614.1 | 490.4±1867.5 | 53.8±1944.9 | 0.854 |
| Vitamin C (mg) | 84.3±35.8 | 147.6±210.4 | 63.2±216.2 | 0.056 |
| Calcium (mg) | 794.1±258.1 | 622.6±258.6 | -171.5±237.5 | **<0.001** |
| Iron (mg) | 13.3±5.5 | 14.3±10.2 | 0.9±1.0 | 0.528 |
| Vitamin D (μg) | 1.3±1.1 | 0.8±2.1 | -0.5±2.1 | 0.118 |
| Alpha-Tocopherol (mg) | 10.9±4.4 | 10.9±15.7 | 0.0±16.4 | 0.996 |
| Folate (Total) (μg) | 330.0±123.9 | 422.0±312.4 | 92.1±307.9 | **0.051** |
| Magnesium (mg) | 289.6±93.6 | 384.0±348.5 | 94.5±339.8 | 0.069 |

^a^ *p*-value was derived from a paired sample t-test.

The numbers in bold are statistically significant (*p*-value ≤ 0.05).
